# Characterization of plant growth-promoting rhizobacteria (PGPR) in Persian walnut associated with drought stress tolerance

**DOI:** 10.1038/s41598-022-16852-6

**Published:** 2022-07-26

**Authors:** Naser Lotfi, Ali Soleimani, Ramazan Çakmakçı, Kourosh Vahdati, Parisa Mohammadi

**Affiliations:** 1 Department of Horticulture, Faculty of Agriculture, University of Zanjan, Zanjan, Iran; 2 Department of Agronomy, Faculty of Agriculture, Çanakkale Onsekiz Mart Üniversitesi Çomü, Çanakkale, Turkey; 3 Department of Horticulture, College of Aburaihan, University of Tehran, Tehran, Iran; 4 Department of Plant Protection, Faculty of Agriculture, Ataturk University, Erzurum, Turkey

**Keywords:** Microbiology, Plant sciences

## Abstract

There is a lack of information on the rhizosphere of nut-bearing trees where microbial populations can benefit roots and tree growth. The current research aimed at discovering plant growth-promoting rhizobacteria (PGPR) in the rhizosphere of soil samples from around the root zone of six walnut trees, each of which was considered as a genotype, i.e. ‘TT1’, ‘TT2’, ‘SS2’, ‘ZM1’, ‘Chandler’ and ‘Haward’. The trees grew in different arid and semiarid regions of Iran and Turkey. The strains were isolated and identified based on different morphological and biochemical markers. Drought-stress tolerance was assessed in the case of each isolate through their transfer to culture medium, containing polyethylene glycol (PEG_6000_) at 0 and 373.80 g L^−1^. Resilient strains were analyzed for measuring their ability to produce siderophore, hydrogen cyanide (HCN), Indole-3-acetic acid (IAA) and Gibberellic acid (GA_3_). In sum, 211 isolates were identified, of which a large number belonged to the *Bacillus* genus and, specifically, 78% of the strains were able to grow under drought stress conditions. The genus *Arthrobacter* was only detected in the rhizosphere of ‘ZM1’, ‘Haward’ and ‘TT1’ genotypes. In 4% of the strains, IAA production exceeded 53 mg L^−1^, while a high level of phosphorus solubility was verified in 6% of the strains. No strain was found to have the capability of producing HCN. The strains were screened for drought-tolerance, which resulted in the discovery of two promising strains, i.e. ZM39 and Cha43. Based on molecular identification through amplification and sequencing of the 16S rDNA gene, these two strains seemed to belong to *Bacillus velezensis* and *Bacillus amyloliquefaciens,* respectively. The discovery of new PGPR strains could probably assist walnut trees in improving their mechanisms of adaptation to drought stress.

## Introduction

Drought stress is a pervasive impediment to horticultural endeavors in many regions of the world. Previous studies have shown that abiotic stress conditions, such as drought or flooding, can adversely affect the root microbiome, root exudates and morp^1,2^, thereby affecting plant growth and productivity. Among the many solutions that can assist in countering drought stress, the inoculation of rhizosphere with PGPR can have a wide range of benefits for plant roots and for the maintenance of plant growth in dry environments. In general, PGPRs can produce siderophores, antibiotics and cyanides that can further increase the availability of soil nutrients, stabilize atmospheric nitrogen, and generate antagonistic activity against plant pathogens and harmful microbes in the soil^3,4^.

The rhizosphere is a narrow zone of soil that encircles plant roots. It is a rich site of nutrients where a variety of organic compounds are released from the roots by exudation, secretion and deposition. This ecological feature of the rhizosphere provides carbon and energy sources to microorganisms, thereby facilitating a rapid rate of microbial growth and activity that can benefit the roots. While PGPR communities are able to colonize roots and stimulate plant growth^5^, it is possible to inoculate the rhizosphere artificially with more of the relevant PGPRs and increase their populations in the rhizosphere, thereby increasing the occurrence of benefits for plant roots.

PGPRs are diverse in genera and species, and specific set of PGPRs is usually found in the rhizosphere of each plant species. This means that not all PGPRs are suitable for artificial inoculation in the rhizosphere of a particular plant species. Thus, the correct species of PGPRs in the rhizosphere of a plant species need to be identified first. Then, they can be inoculated artificially in the rhizosphere so as to add to the existing population of PGPRs which naturally exist there. New PGPRs are increasingly being identified among bacterial genera such as *Azospirillum, Pseudomonas, Klebsiella, Azotobacter, Alcaligenes, Enterobacter, Arthrobacter, Bacillus, Serratia*, and *Burkholderia*^6^. With the exception of a few reports, the available literature on the rhizosphere of nut-bearing trees, such as walnut, is not substantive^7,8^.

Persian walnut (*Juglans regia* L.) is a deciduous tree species that has a high degree of adaptability to different growth conditions, although it grows more successfully in temperate regions and can survive at altitudes as high as 3550 m above sea level^9,10^. Walnut trees show allelopathy effects, meaning that unique species of microorganisms can survive and coexist under the canopy of the walnut trees. Therefore, it appears that microorganisms in the rhizosphere of this tree are entirely specific and different from those of other tree species. Yu et al.^11^ identified 11 strains with phosphate-solubilizing activity in the walnut rhizosphere using 16S rDNA. These 11 strains belonged to five genera namely, *Pseudomonas*, *Staphylococcus*, *Planmicrobium*, *Microbacterium* and *Acinetobacter*^11^.

Although few PGPRs have been inoculated in the rhizosphere of walnut roots so far, the benefits of inoculation are considered valuable in the available literature^7,12^, including research on the mitigation of drought stress and maintenance of biological features which can be achieved by inoculating the correct species of PGPRs in the rhizosphere. Accordingly, a complete and detailed identification of PGPR communities in arid and semiarid habitats of walnut can assist with tolerance to drought stress conditions. In this regard, the available literature can benefit from new research on reliable comparisons between the specific abilities of each identified strain, especially in terms of their ability to withstand unfavorable conditions of stress^13^. Such cases of research can contribute to their preservation and mass production by high-tech facilities in approaching future exploitation as biofertilizers.

Since walnut trees have the tolerance to grow in adverse conditions, it was hypothesized that it coexists with PGPRs that contribute to its resilient growth behavior. The present study was conducted to explore and identify bacterial diversity in the rhizosphere of several promising walnut genotypes grown in arid and semiarid regions of Iran. A thorough comparison was carried out among the identified and isolated strains under drought stress. Definitely, resilient strains would be considered as resources for improving walnut production in regions where drought stress is prevalent.

## Materials and methods

### Plant materials for rhizosphere sampling

The population of each strain was collected from the rhizosphere surrounding the root zone in each of six walnut genotypes, i.e. ‘TT1’, ‘TT2’, ‘SS2’, ‘ZM1’, ‘Chandler’ and ‘Haward’, grown in arid and semiarid regions of Iran and Turkey. These genotypes were coded nominally by the authors and are free to access upon request. All experiments were performed in accordance with relevant guidelines and regulations. The phenological and agronomic traits of the walnut genotypes were provided as supplementary data (Table S1).

### Sample collection and isolation of PGPR

In the case of each genotype, soil samples were collected from 5 to 50 cm depths of the rhizosphere around the walnut roots. The samples were coded as ‘RS-SS2’, ‘RS-TT2’, ‘RS-TT1’, ‘RS-ZM1’, ‘RS-Haward’ and ‘RS-Chandler’, and were placed in sterilized plastic bags before being transferred to the laboratory after 24 h. The samples were stored at 4 °C until further analysis followed procedures that were outlined in a soil test-kit model (KA-054). Bacterial isolation involved adding 1 g of the rhizosphere soil to 9 ml of phosphate buffer with a specificity of 20 mM, pH 7.0, which was incubated inside a rotary shaker at 150 rpm for 30 min at 30 °C. The strains were grown in nutrient agar culture media (NA) and stored in a nutrient broth (NB) containing 15% glycerol at 80 °C, which is a long-term storage method for these bacteria.

### Morphological and biochemical characterization

The strains were identified in relation to biochemical and morphological traits. Morphological characteristics were identified by observing each bacterial colony. A composite microscope (Gaynor) at 100X was used for observing the color, shape, size and margin of colonies of the bacterium. Also, the cell shape, size, endospore presence and the gram’s reaction were considered. Several biochemical traits were evaluated according to the Manual of Bergey’s Determinative Bacteriology^14^.

### Drought stress tolerance assessment for the isolates

Single colonies were cultured and transplanted into 500 ml containers containing NB. They were aerobically grown on a rotary vibrator at 150 rpm for 48 h at 27 °C. This was followed by adding distilled water along with a suspension of bacteria, reaching a final concentration of 10^9^ ml mL^−1^. Various strains were transferred to a culture medium containing PEG_6000_ at 0 and 373.80 g L^−1^ which was equivalent to osmotic stress at 0 and − 1.5 MPa. The growth rate of isolates in a pilot study was previously recorded at different PEG_6000_ concentrations. Screening tests on these strains were calculated based on the growth rate of bacteria by measuring the optical density of their growth medium at 600 nm, using spectrophotometric analysis. Cultures that were able to grow in the presence of PEG_6000_ were analyzed further for biochemical traits through a factorial (PEG_6000_ at 0 and 373.80 g L^−1^ as factors) based on completely random design.

### Phosphate-solubilization and amylase activity assay

The culture medium in this test was a modified type of the Sperber (Sp) medium^15^, and was supplemented with inositol hexaphosphate. Instead of tricalcium phosphate; however, inositol hexaphosphate was used. The test was performed on both solid and liquid media. The Sp medium, contained 2.5 g L^−1^ calcium phytate, was distributed in petri dishes under sterile conditions. Each petri dish was divided into six equal parts and the different fresh isolates were cultured by the dipping method in a separate petri dish, with three replicates, and stored in an incubator at 28 °C. The colony diameter grew, leaving different diameters of the transparent halos from the dissolution of the phosphate around each colony. The diameters were measured at intervals of 1, 2, 4, 8 and 10 days, and then the diameter of the halo was measured against the colony diameter in each isolate. Isolates which had halo-to-colony diameter ratios of more than 1.5 mm were evaluated by culture methods on liquid medium. At this stage, the modified Sp medium of 2.5 g L^−1^ calcium phytate was used. Phosphorus was measured in inoculated and control (un-inoculated) media. The preparation of the standard curve was similar to the mineral method. Amylase activity was performed using a modified version of Nack-Moon et al.^16^ method. After incubation at 30 °C for 5 days, the production of amylase, which was detected using soluble starch (1%), was screened by adding iodine to the culture. Liquid starch medium was used as control. Discoloration confirmed the presence of amylase production of bacterial isolates that decompose starch.

### Siderophore and hydrogen cyanide production

The CAS method^17^ was used for quantitative measurement of siderophore produced by the strains. The reaction mixture included a cell-free extract of supernatant (0.1 ml) which was mixed with 0.5 ml of the CAS assay solution along with 10 μl of a shuttle solution (0.2 M 5-sulfosalicylic acid). The reaction mixture was stored at room temperature for 10 min, and then the absorbance was measured at 630 nm using a UV–VIS spectrophotometer (SL164, Systronics). For the control treatment, all of the compounds were used, except the extract which was obtained from the cell. Each siderophore unit was calculated using the following formula:

$$\mathrm{Siderophore \ Unit }({\%})=\frac{\left(Ar-As\right)}{As}\times 100$$

where *Ar* is the absorbance at 630 nm of reference (CAS assay solution + uninoculated media), and *As* is the absorbance at 630 nm of the sample (CAS assay solution + supernatant).

Production of HCN was done using a nutrient agar medium containing 0.44% glycine^18^. The surface of the agar was streaked with one-day culture and was coated with Whatman No. 1 filter paper. It was immersed in 2% sodium carbonate solution and 0.5% picric acid. This was followed by storage at 30 °C for 72 h. The changes observed in the color of the filter paper ranged from yellow to orange, red and brown. These colors indicate respectively the low, medium and high levels of HCN production by the strains. A quantitative analysis of HCN was done by analyzing strips of filter paper, which were changed by picric acid and sodium bicarbonate. After inoculating the tubes and keeping them at 30 °C for seven days, the strips were stored in double-distilled water and the changes in color were observed at 625 nm.

### Estimations of Indole-3-acetic acid and gibberellins

Primarily, bacterial isolates were grown in NB for 72 h at 37 °C in a shaker. IAA was eluted by methanol according to a method described by Żur et al.^19^. The supernatant comprised formic acid (0.1%) aqueous solution (solvent A) and acetonitrile: methanol (1:1) mixture (solvent B). The MRM (Multiple Reaction Monitoring) mode was used for monitoring each analyzed compound in which the most abundant ion product functioned as the quantifier. Also, another abundant ion product was used for identifying the phytohormones. For the extraction of gibberellic acid (GA_3_), extracts were prepared with a mixture of iso-propanol/H_2_O/concentrated HCl (2:1:0.002, v:v:v). Then, they were centrifuged and purified within a series of steps. A re-dissolved operation was performed with a final concentration in 100 L methanol. Half of the volume was subjected to an ESI-triple quadrupole mass spectrometer device (HPLC-ESI-MS/MS, Applied Biosystems, USA) which was equipped with a reverse-phase C18 Gemini column (150 9 2.00 mm, 5-lm particle size, Phenomenex, USA)^20^.

### Molecular identification of the bacterial strains

Two strains were then selected for further molecular characterization and identification. For this purpose, amplification occurred through PCR reaction, followed by subsequent sequencing of the 16S rDNA gene. The cultures were grown in LB for DNA extraction at 26 °C on a shaker (250 rpm). DNA was extracted using the DNeasy Tissue Kit (Qiagen). DNA concentration and quality were determined using a spectrophotometer and by gel electrophoresis on agarose (1%). The thermal cycles of the PCR operated according to a protocol used by Rees and Li, 2004. The amplified products were sent to Macrogen (a South Korean company) for sequencing the samples in both directions. The NCBI database was used as a platform for the comparison of sequence data by the BLAST program. The sequences of representative strains were submitted to the GenBank database and accession numbers were obtained.

## Results and discussion

The physical and chemical characteristics of soil samples from the rhizosphere of each genotype differed from another (Table 1). In total, 205 non-identical bacteria were found in the rhizosphere surrounding the walnut roots (Table 2). Morpho-biochemical detection methods revealed that 76, 67, 44 and 18 isolates belonged to *Micrococcus*, *Bacillus, Pseudomonas* and *Arthrobacter*, respectively. The genus *Arthrobacter* was only detected in the rhizospheres of ‘ZM1’, ‘Haward’ and ‘TT1’ genotypes. The rhizosphere of the ‘RS-ZM1’ genotype contained the highest number of isolates, i.e. 19, 12, 15 and 2 of *Bacillus, Pseudomonas, Micrococcus* and *Arthrobacter* genera, respectively, whereas the rhizosphere of the ‘RS-TT2’ genotype consisted of the least number of isolates, i.e. 6, 7, 9 and 0 of *Bacillus, Pseudomonas, Micrococcus* and *Arthrobacter* genera, respectively (Table 2). Rhizobacteria with PGP-activity are known to occur in several bacterial phyla (*Firmicutes, Proteobacteria* and *Actinobacteria*), including strains that belong to the genera *Azospirillum, Bacillus, Azotobacter, Agrobacterium, Alcaligens, Pseudomonas, Arthobacter, Comamonas, Burkholde-ria, Rhizobium, Pantoea, Variovorax* and *Serratia*^21^. In confirming a large range of diversity among the bacterial strains, our results were in agreement with previous findings reported by Vega et al.^22^ in a research that led to the isolation of large numbers of bacterial strains from the rhizosphere of coffee plants.

**Table 1:** Physio-chemical characteristics and mineral elements of soils from the different rhizospheres of walnut trees.

Rhizosphere soil samples	Sand	Clay	Silt	AECC	OC	FC	pH	EC	N	P	K	Fe	Mn	Cu	Zn	Br	Mg	Na	Cl
	* (g/100 g soil)							(dS/m)	(mg/kg)										
‘RS-ZM1’	36.85	20.45	42.70	6.38	1.12	23.28	8.12	2.89	0.84	12.37	402.31	6.51	8.92	1.34	0.82	0.10	5.62	5.79	9.32
‘RS-SS2’	38.42	28.91	32.60	4.37	0.62	21.08	7.68	1.45	0.21	4.37	306.24	2.34	4.76	0.58	0.27	0.03	4.38	2.75	4.21
‘RS-TT2’	44.82	25.94	29.20	4.38	0.73	20.16	7.72	1.57	0.63	7.92	256.35	3.24	3.73	0.42	0.31	0.08	6.81	4.16	1.82
‘RS-Haward’	41.82	25.31	32.87	5.46	0.94	22.11	7.64	2.05	0.42	8.40	336.58	4.48	6.70	0.98	0.44	0.09	3.85	3.46	5.15
‘RS-TT1’	37.18	34.61	28.20	4.68	0.71	20.13	7.62	1.43	0.57	11.03	284.39	5.91	4.52	0.61	0.57	0.10	4.69	2.15	4.37
‘RS-Chandler’	35.62	34.63	29.70	6.72	1.24	24.38	8.12	2.27	2.37	14.38	367.42	8.37	7.91	0.92	1.13	0.14	5.39	4.61	9.37

*AECC* active equivalent calcium carbonate, *OC* organic carbon, *FC* field capacity, *pH* acidity of soil, *EC* electrical conductivity.

*Each measurement is the mean of three replications. All of the nutrients were measured as the absorbable form.

**Table 2:** Morpho-biochemical characterization of the rhizobacteria isolates from different rhizospheres of walnut trees.

Rhizosphere soil samples	Related isolates	Gram test	Catalase	Oxidase	Sucrose	Nitrate reduction	Levan	Citrate	Mobility	Aerobic	Anaerobic	H_2_S production	Indole test	Methyl red test	Voges-Proskauer test	Starch hydrolysis	Cell shape	Endospore position	Probable genus
‘RS-ZM1’	ZM1	−	+	+	+	+	−	+	+	+	−	+	+	+	+	−	Rod shape	−	*Pseudomonas*
	ZM2	−	+	+	+	+	−	+	+	+	−	+	+	+	+	−	Rod shape	−	*Pseudomonas*
	ZM3	−	−	+	+	+	+	−	+	+	+	+	−	+	+	−	Rod shape	−	*Pseudomonas*
	ZM4	+	+	+	−	+	+	−	+	+	−	−	−	−	+	+	Long rods	Central	*Bacillus*
	ZM5	+	+	+	−	+	+	−	−	+	+	−	−	−	+	+	Long rods	Central	*Bacillus*
	ZM6	+	+	+	−	+	+	−	−	+	+	−	−	+	+	+	Long rods	Central	*Bacillus*
	ZM7	+	+	+	−	+	+	−	−	+	−	−	−	+	+	+	Medium rods	Central	*Bacillus*
	ZM8	+	+	+	−	+	+	−	−	+	−	−	−	−	+	+	Long rods	Central	*Bacillus*
	ZM9	+	+	+	−	+	+	−	−	+	+	−	−	−	+	+	Medium rods	Central	*Bacillus*
	ZM10	−	+	+	+	+	−	+	+	+	−	+	+	+	+	−	Rod shape	−	*Pseudomonas*
	ZM11	−	+	+	+	+	+	−	+	+	−	+	−	+	+	−	Rod shape	−	*Pseudomonas*
	ZM12	−	−	+	+	+	+	−	+	+	+	+	−	+	+	−	Rod shape	−	*Pseudomonas*
	ZM13	+	+	+	+	−	+	−	+	+	−	−	−	−	−	+	Minute cocci	−	*Micrococcus*
	ZM14	+	+	+	+	−	+	−	+	+	−	−	−	−	−	+	Minute cocci	−	*Micrococcus*
	ZM15	+	+	+	+	−	+	−	+	+	−	−	−	−	−	+	Minute cocci	−	*Micrococcus*
	ZM16	+	+	+	+	−	+	−	+	+	−	−	−	−	−	+	Minute cocci	−	*Micrococcus*
	ZM17	+	+	+	+	−	+	−	+	−	+	−	−	−	−	+	Minute cocci	−	*Micrococcus*
	ZM18	+	+	+	−	+	+	−	−	+	+	−	−	−	+	+	Medium rods	Central	*Bacillus*
	ZM19	+	+	+	−	+	+	−	−	+	+	−	−	+	+	+	Long rods	Central	*Bacillus*
	ZM20	−	+	+	+	+	−	+	+	+	−	+	+	+	+	−	Rod shape	−	*Pseudomonas*
	ZM21	−	−	+	+	+	+	−	+	+	+	+	−	+	+	−	Rod shape	−	*Pseudomonas*
	ZM22	+	+	+	+	−	+	−	+	+	−	−	−	−	−	+	Minute cocci	−	*Micrococcus*
	ZM23	+	+	+	+	−	+	−	+	+	−	−	−	−	−	+	Minute cocci	−	*Micrococcus*
	ZM24	+	+	+	+	−	+	−	+	−	+	−	−	−	−	+	Minute cocci	−	*Micrococcus*
	ZM25	+	+	+	−	+	+	−	−	+	+	−	−	−	+	+	Long rods	Central	*Bacillus*
	ZM26	+	+	+	−	+	+	−	−	+	+	−	−	+	+	+	Medium rods	Central	*Bacillus*
	ZM27	+	+	+	−	+	+	−	−	+	−	−	−	+	+	+	Long rods	Central	*Bacillus*
	ZM28	+	+	+	−	+	+	−	−	+	−	−	−	−	+	+	Medium rods	Central	*Bacillus*
	ZM29	+	+	+	−	+	+	−	−	+	+	−	−	−	+	+	Long rods	Central	*Bacillus*
	ZM30	+	+	+	+	−	+	−	+	+	−	−	−	−	−	+	Minute cocci	−	*Micrococcus*
	ZM31	+	+	+	+	−	+	−	+	+	−	−	−	−	−	+	Minute cocci	−	*Micrococcus*
	ZM32	+	+	+	+	−	+	−	+	+	−	−	−	−	−	+	Minute cocci	−	*Micrococcus*
	ZM33	+	+	+	−	+	+	−	−	+	+	−	−	+	+	+	Long rods	Central	*Bacillus*
	ZM34	+	+	+	−	+	+	−	−	+	−	−	−	+	+	+	Long rods	Central	*Bacillus*
	ZM35	+	+	+	−	+	+	−	−	+	−	−	−	−	+	+	Long rods	Central	*Bacillus*
	ZM36	+	+	+	−	+	+	−	−	+	+	−	−	−	+	+	Long rods	Central	*Bacillus*
	ZM37	+	+	+	+	−	+	−	+	+	−	−	−	−	−	+	Minute cocci	−	*Micrococcus*
	ZM38	+	+	+	+	−	+	−	+	−	+	−	−	−	−	+	Minute cocci	−	*Micrococcus*
	ZM39	+	+	+	−	+	+	−	−	+	+	−	−	−	+	+	Long rods	Central	*Bacillus*
	ZM40	+	+	+	−	+	+	−	−	+	+	−	−	+	+	+	Long rods	Central	*Bacillus*
	ZM41	−	+	+	+	+	−	+	+	+	−	+	+	+	+	−	Rod shape	−	*Pseudomonas*
	ZM42	−	−	+	+	+	+	−	+	+	+	+	−	+	+	−	Rod shape	−	*Pseudomonas*
	ZM43	+	+	−	−	−	+	−	+	+	−	−	−	+	+	−	Rod and cocci	−	*Arthrobacter*
	ZM44	+	+	−	−	−	+	−	+	+	−	−	+	−	+	−	Rod and cocci	−	*Arthrobacter*
	ZM45	+	+	+	+	−	+	−	+	+	−	−	−	−	−	+	Minute cocci	−	*Micrococcus*
	ZM46	+	+	+	+	−	+	−	+	+	−	−	−	−	−	+	Minute cocci	−	*Micrococcus*
	ZM47	−	+	+	+	+	−	+	+	+	−	+	+	+	+	−	Rod shape	−	*Pseudomonas*
	ZM48	−	+	+	+	+	+	−	+	+	−	+	−	+	+	−	Rod shape	−	*Pseudomonas*
‘RS-SS2’	SS21	−	−	+	+	+	+	−	+	+	+	+	−	+	+	−	Rod shape	−	*Pseudomonas*
	SS22	+	+	+	−	+	+	−	−	+	+	−	−	−	+	+	Long rods	Central	*Bacillus*
	SS23	+	+	+	−	+	+	−	−	+	+	−	−	+	+	+	Medium rods	Central	*Bacillus*
	SS24	+	+	+	−	+	+	−	−	+	−	−	−	+	+	+	Long rods	Central	*Bacillus*
	SS25	+	+	+	+	−	+	−	+	+	−	−	−	−	−	+	Minute cocci	−	*Micrococcus*
	SS26	+	+	+	+	−	+	−	+	−	+	−	−	−	−	+	Minute cocci	−	*Micrococcus*
	SS27	−	+	+	+	+	−	+	+	+	−	+	+	+	+	−	Rod shape	−	*Pseudomonas*
	SS28	−	+	+	+	+	+	−	+	+	−	+	−	+	+	−	Rod shape	−	*Pseudomonas*
	SS29	−	−	+	+	+	+	−	+	+	+	+	−	+	+	−	Rod shape	−	*Pseudomonas*
	SS210	+	+	+	+	−	+	−	+	+	−	−	−	−	−	+	Minute cocci	−	*Micrococcus*
	SS211	+	+	+	+	−	+	−	+	+	−	−	−	−	−	+	Minute cocci	−	*Micrococcus*
	SS212	+	+	+	+	−	+	−	+	+	−	−	−	−	−	+	Minute cocci	−	*Micrococcus*
	SS213	+	+	+	+	−	+	−	+	+	−	−	−	−	−	+	Minute cocci	−	*Micrococcus*
	SS214	+	+	+	+	−	+	−	+	−	+	−	−	−	−	+	Minute cocci	−	*Micrococcus*
	SS215	+	+	+	−	+	+	−	−	+	+	−	−	−	+	+	Long rods	Central	*Bacillus*
	SS216	+	+	+	−	+	+	−	−	+	+	−	−	+	+	+	Long rods	Central	*Bacillus*
	SS217	−	+	+	+	+	−	+	+	+	−	+	+	+	+	−	Rod shape	−	*Pseudomonas*
	SS218	−	−	+	+	+	+	−	+	+	+	+	−	+	+	−	Rod shape	−	*Pseudomonas*
	SS219	+	+	+	+	−	+	−	+	+	−	−	−	−	−	+	Minute cocci	−	*Micrococcus*
	SS220	+	+	+	+	−	+	−	+	+	−	−	−	−	−	+	Minute cocci	−	*Micrococcus*
	SS221	+	+	+	+	−	+	−	+	+	−	−	−	−	−	+	Minute cocci	−	*Micrococcus*
	SS222	+	+	+	+	−	+	−	+	+	−	−	−	−	−	+	Minute cocci	−	*Micrococcus*
	SS223	+	+	+	+	−	+	−	+	+	−	−	−	−	−	+	Minute cocci	−	*Micrococcus*
	SS224	+	+	+	−	+	+	−	−	+	+	−	−	+	+	+	Medium rods	Central	*Bacillus*
	SS225	+	+	+	−	+	+	−	−	+	−	−	−	+	+	+	Medium rods	Central	*Bacillus*
	SS226	+	+	+	−	+	+	−	−	+	−	−	−	−	+	+	Long rods	Central	*Bacillus*
	SS227	+	+	+	−	+	+	−	−	+	+	−	−	−	+	+	Long rods	Central	*Bacillus*
	SS228	+	+	+	+	−	+	−	+	+	−	−	−	−	−	+	Minute cocci	−	*Micrococcus*
	SS229	+	+	+	+	−	+	−	+	−	+	−	−	−	−	+	Minute cocci	−	*Micrococcus*
	SS230	+	+	+	−	+	+	−	−	+	+	−	−	−	+	+	Long rods	Central	*Bacillus*
	SS231	+	+	+	−	+	+	−	−	+	+	−	−	+	+	+	Long rods	Central	*Bacillus*
	SS232	+	+	+	−	+	+	−	−	+	−	−	−	+	+	+	Long rods	Central	*Bacillus*
‘RS-TT2’	TT21	−	−	+	+	+	+	−	+	+	+	+	−	+	+	−	Rod shape	−	*Pseudomonas*
	TT22	+	+	+	+	−	+	−	+	+	−	−	−	−	−	+	Minute cocci	−	*Micrococcus*
	TT23	+	+	+	+	−	+	−	+	+	−	−	−	−	−	+	Minute cocci	−	*Micrococcus*
	TT24	+	+	+	+	−	+	−	+	+	−	−	−	−	−	+	Minute cocci	−	*Micrococcus*
	TT25	+	+	+	+	−	+	−	+	−	+	−	−	−	−	+	Minute cocci	−	*Micrococcus*
	TT26	+	+	+	−	+	+	−	−	+	+	−	−	−	+	+	Long rods	Central	*Bacillus*
	TT27	+	+	+	−	+	+	−	−	+	+	−	−	+	+	+	Long rods	Central	*Bacillus*
	TT28	−	+	+	+	+	−	+	+	+	−	+	+	+	+	−	Rod shape	−	*Pseudomonas*
	TT29	−	−	+	+	+	+	−	+	+	+	+	−	+	+	−	Rod shape	−	*Pseudomonas*
	TT210	+	+	+	−	+	+	−	−	+	−	−	−	−	+	+	Long rods	Central	*Bacillus*
	TT211	+	+	+	−	+	+	−	−	+	+	−	−	−	+	+	Long rods	Central	*Bacillus*
	TT212	+	+	+	+	−	+	−	+	+	−	−	−	−	−	+	Minute cocci	−	*Micrococcus*
	TT213	+	+	+	+	−	+	−	+	+	−	−	−	−	−	+	Minute cocci	−	*Micrococcus*
	TT214	+	+	+	+	−	+	−	+	+	−	−	−	−	−	+	Minute cocci	−	*Micrococcus*
	TT215	−	+	+	+	+	−	+	+	+	−	+	+	+	+	−	Rod shape	−	*Pseudomonas*
	TT216	−	+	+	+	+	+	−	+	+	−	+	−	+	+	−	Rod shape	−	*Pseudomonas*
	TT217	+	+	+	+	−	+	−	+	+	−	−	−	−	−	+	Minute cocci	−	*Micrococcus*
	TT218	+	+	+	+	−	+	−	+	−	+	−	−	−	−	+	Minute cocci	−	*Micrococcus*
	TT219	+	+	+	−	+	+	−	−	+	+	−	−	−	+	+	Long rods	Central	*Bacillus*
	TT220	+	+	+	−	+	+	−	−	+	+	−	−	+	+	+	Long rods	Central	*Bacillus*
	TT221	−	+	+	+	+	−	+	+	+	−	+	+	+	+	−	Rod shape	−	*Pseudomonas*
	TT222	−	−	+	+	+	+	−	+	+	+	+	−	+	+	−	Rod shape	−	Pseudomonas
‘RS-Haward’	Haw1	+	+	+	+	−	+	−	+	+	−	−	−	−	−	+	Minute cocci	−	*Micrococcus*
	Haw2	+	+	+	+	−	+	−	+	+	−	−	−	−	−	+	Minute cocci	−	*Micrococcus*
	Haw3	+	+	+	+	−	+	−	+	−	+	−	−	−	−	+	Minute cocci	−	*Micrococcus*
	Haw4	+	+	+	−	+	+	−	−	+	+	−	−	−	+	+	Medium rods	Central	*Bacillus*
	Haw5	+	+	+	−	+	+	−	−	+	+	−	−	+	+	+	Long rods	Central	*Bacillus*
	Haw6	−	+	+	+	+	−	+	+	+	−	+	+	+	+	−	Rod shape	−	*Pseudomonas*
	Haw7	−	−	+	+	+	+	−	+	+	+	+	−	+	+	−	Rod shape	−	*Pseudomonas*
	Haw8	+	+	+	+	−	+	−	+	+	−	−	−	−	−	+	Minute cocci	−	*Micrococcus*
	Haw9	−	+	+	+	+	−	+	+	+	−	+	+	+	+	−	Rod shape	−	*Pseudomonas*
	Haw10	−	−	+	+	+	+	−	+	+	+	+	−	+	+	−	Rod shape	−	*Pseudomonas*
	Haw11	+	+	+	+	−	+	−	+	+	−	−	−	−	−	+	Minute cocci	−	*Micrococcus*
	Haw12	+	+	+	+	−	+	−	+	+	−	−	−	−	−	+	Minute cocci	−	*Micrococcus*
	Haw13	+	+	+	+	−	+	−	+	+	−	−	−	−	−	+	Minute cocci	−	*Micrococcus*
	Haw14	+	+	+	+	−	+	−	+	+	−	−	−	−	−	+	Minute cocci	−	*Micrococcus*
	Haw15	+	+	+	+	−	+	−	+	+	−	−	−	−	−	+	Minute cocci	−	*Micrococcus*
	Haw16	+	+	+	−	+	+	−	−	+	−	−	−	−	+	+	Long rods	Central	*Bacillus*
	Haw17	+	+	+	−	+	+	−	−	+	+	−	−	−	+	+	Long rods	Central	*Bacillus*
	Haw18	+	+	+	+	−	+	−	+	+	−	−	−	−	−	+	Minute cocci	−	*Micrococcus*
	Haw19	+	+	+	+	−	+	−	+	−	+	−	−	−	−	+	Minute cocci	−	*Micrococcus*
	Haw20	+	+	+	−	+	+	−	−	+	+	−	−	−	+	+	Long rods	Central	*Bacillus*
	Haw21	+	+	+	−	+	+	−	−	+	+	−	−	+	+	+	Long rods	Central	*Bacillus*
	Haw22	−	+	+	+	+	−	+	+	+	−	+	+	+	+	−	Rod shape	−	*Pseudomonas*
	Haw23	−	−	+	+	+	+	−	+	+	+	+	−	+	+	−	Rod shape	−	*Pseudomonas*
	Haw24	+	+	−	−	−	+	−	+	+	−	−	−	+	+	−	Rod and cocci	−	*Arthrobacter*
	Haw25	+	+	−	−	−	+	−	+	+	−	−	+	−	+	−	Rod and cocci	−	*Arthrobacter*
	Haw26	+	+	+	+	−	+	−	+	+	−	−	−	−	−	+	Minute cocci	−	*Micrococcus*
	Haw27	+	+	+	+	−	+	−	+	+	−	−	−	−	−	+	Minute cocci	−	*Micrococcus*
	Haw28	−	+	+	+	+	−	+	+	+	−	+	+	+	+	−	Rod shape	−	*Pseudomonas*
	Haw29	−	+	+	+	+	+	−	+	+	−	+	−	+	+	−	Rod shape	−	*Pseudomonas*
	Haw30	−	−	+	+	+	+	−	+	+	+	+	−	+	+	−	Rod shape	−	*Pseudomonas*
	Haw31	+	+	−	−	−	+	−	+	+	−	−	−	+	+	−	Rod and cocci	−	*Arthrobacter*
	Haw32	+	+	−	−	−	+	−	+	+	−	−	+	−	+	−	Rod and cocci	−	*Arthrobacter*
	Haw33	+	+	−	−	−	+	−	+	+	−	−	−	+	+	−	Rod and cocci	−	*Arthrobacter*
	Haw34	+	+	−	−	−	+	−	+	+	−	−	+	−	+	−	Rod and cocci	−	*Arthrobacter*
	Haw35	+	+	−	−	−	+	−	+	+	−	−	−	+	+	−	Rod and cocci	−	*Arthrobacter*
	Haw36	+	+	−	−	−	+	−	+	+	−	−	+	−	+	−	Rod and cocci	−	*Arthrobacter*
	Haw37	+	+	+	−	+	+	−	−	+	+	−	−	−	+	+	Long rods	Central	*Bacillus*
	Haw38	+	+	+	−	+	+	−	−	+	+	−	−	+	+	+	Long rods	Central	*Bacillus*
	Haw39	−	+	+	+	+	−	+	+	+	−	+	+	+	+	−	Rod shape	−	*Pseudomonas*
	Haw40	−	−	+	+	+	+	−	+	+	+	+	−	+	+	−	Rod shape	−	*Pseudomonas*
	Haw41	+	+	+	−	+	+	−	−	+	+	−	−	−	+	+	Long rods	Central	*Bacillus*
	Haw42	+	+	+	−	+	+	−	−	+	+	−	−	+	+	+	Long rods	Central	*Bacillus*
‘RS-TT1’	TT11	+	+	+	−	+	+	−	−	+	+	−	−	−	+	+	Medium rods	Central	*Bacillus*
	TT12	+	+	+	−	+	+	−	−	+	+	−	−	+	+	+	Medium rods	Central	*Bacillus*
	TT13	−	+	+	+	+	−	+	+	+	−	+	+	+	+	−	Rod shape	−	*Pseudomonas*
	TT14	−	−	+	+	+	+	−	+	+	+	+	−	+	+	−	Rod shape	−	*Pseudomonas*
	TT15	+	+	+	+	−	+	−	+	+	−	−	−	−	−	+	Minute cocci	−	*Micrococcus*
	TT16	+	+	+	+	−	+	−	+	+	−	−	−	−	−	+	Minute cocci	−	*Micrococcus*
	TT17	+	+	+	+	−	+	−	+	+	−	−	−	−	−	+	Minute cocci	−	*Micrococcus*
	TT18	+	+	+	+	−	+	−	+	+	−	−	−	−	−	+	Minute cocci	−	*Micrococcus*
	TT19	+	+	+	+	−	+	−	+	+	−	−	−	−	−	+	Minute cocci	−	*Micrococcus*
	TT110	+	+	+	−	+	+	−	−	+	+	−	−	+	+	+	Long rods	Central	*Bacillus*
	TT111	+	+	+	−	+	+	−	−	+	−	−	−	+	+	+	Long rods	Central	*Bacillus*
	TT112	+	+	+	−	+	+	−	−	+	−	−	−	−	+	+	Medium rods	Central	*Bacillus*
	TT113	+	+	+	−	+	+	−	−	+	+	−	−	−	+	+	Long rods	Central	*Bacillus*
	TT114	+	+	+	+	−	+	−	+	+	−	−	−	−	−	+	Minute cocci	−	*Micrococcus*
	TT115	+	+	+	+	−	+	−	+	−	+	−	−	−	−	+	Minute cocci	−	*Micrococcus*
	TT116	+	+	+	−	+	+	−	−	+	+	−	−	−	+	+	Long rods	Central	*Bacillus*
	TT117	+	+	+	−	+	+	−	−	+	+	−	−	+	+	+	Long rods	Central	*Bacillus*
	TT118	+	+	+	−	+	+	−	−	+	−	−	−	+	+	+	Long rods	Central	*Bacillus*
	TT119	−	−	+	+	+	+	−	+	+	+	+	−	+	+	−	Rod shape	−	*Pseudomonas*
	TT120	+	+	+	+	−	+	−	+	+	−	−	−	−	−	+	Minute cocci	−	*Micrococcus*
	TT121	+	+	+	+	−	+	−	+	+	−	−	−	−	−	+	Minute cocci	−	*Micrococcus*
	TT122	+	+	+	+	−	+	−	+	+	−	−	−	−	−	+	Minute cocci		*Micrococcus*
	TT123	+	+	+	+	−	+	−	+	−	+	−	−	−	−	+	Minute cocci		*Micrococcus*
	TT124	+	+	−	−	−	+	−	+	+	−	−	+	−	+	−			*Arthrobacter*
	TT125	+	+	−	−	−	+	−	+	+	−	−	−	+	+	−	-	−	*Arthrobacter*
‘RS-Chandler’	Cha10	−	−	+	+	+	+	−	+	+	+	+	−	+	+	−	Rod shape	−	*Pseudomonas*
	Cha12	+	+	+	+	−	+	−	+	+	−	−	−	−	−	+	Minute cocci	−	*Micrococcus*
	Cha13	+	+	+	+	−	+	−	+	+	−	−	−	−	−	+	Minute cocci	−	*Micrococcus*
	Cha14	+	+	+	+	−	+	−	+	−	+	−	−	−	−	+	Minute cocci	−	*Micrococcus*
	Cha15	+	+	+	−	+	+	−	−	+	+	−	−	−	+	+	Long rods	Central	*Bacillus*
	Cha16	+	+	+	−	+	+	−	−	+	+	−	−	+	+	+	Long rods	Central	*Bacillus*
	Cha17	−	+	+	+	+	−	+	+	+	−	+	+	+	+	−	Rod shape	−	*Pseudomonas*
	Cha18	−	−	+	+	+	+	−	+	+	+	+	−	+	+	−	Rod shape	−	*Pseudomonas*
	Cha19	+	+	+	+	−	+	−	+	+	−	−	−	−	−	+	Minute cocci	−	*Micrococcus*
	Cha20	−	+	+	+	+	−	+	+	+	−	+	+	+	+	−	Rod shape	−	*Pseudomonas*
	Cha21	−	−	+	+	+	+	−	+	+	+	+	−	+	+	−	Rod shape	−	*Pseudomonas*
	Cha22	+	+	+	+	−	+	−	+	+	−	−	−	−	−	+	Minute cocci	−	*Micrococcus*
	Cha23	+	+	+	+	−	+	−	+	+	−	−	−	−	−	+	Minute cocci	−	*Micrococcus*
	Cha24	+	+	+	+	−	+	−	+	+	−	−	−	−	−	+	Minute cocci	−	*Micrococcus*
	Cha25	+	+	+	+	−	+	−	+	+	−	−	−	−	−	+	Minute cocci	−	*Micrococcus*
	Cha26	+	+	+	+	−	+	−	+	+	−	−	−	−	−	+	Minute cocci	−	*Micrococcus*
	Cha27	+	+	+	−	+	+	−	−	+	−	−	−	−	+	+	Long rods	Central	*Bacillus*
	Cha28	+	+	+	−	+	+	−	−	+	+	−	−	−	+	+	Long rods	Central	*Bacillus*
	Cha29	+	+	+	+	−	+	−	+	+	−	−	−	−	−	+	Minute cocci	−	*Micrococcus*
	Cha30	+	+	+	+	−	+	−	+	−	+	−	−	−	−	+	Minute cocci	−	*Micrococcus*
	Cha31	+	+	+	−	+	+	−	−	+	+	−	−	−	+	+	Long rods	Central	*Bacillus*
	Cha32	+	+	+	−	+	+	−	−	+	+	−	−	+	+	+	Long rods	Central	*Bacillus*
	Cha33	−	+	+	+	+	−	+	+	+	−	+	+	+	+	−	Rod shape	−	*Pseudomonas*
	Cha34	−	+	+	+	+	+	−	+	+	−	+	−	+	+	−	Rod shape	−	*Pseudomonas*
	Cha35	−	−	+	+	+	+	−	+	+	+	+	−	+	+	−	Rod shape	−	*Pseudomonas*
	Cha36	+	+	−	−	−	+	−	+	+	−	−	−	+	+	−	Rod and cocci	−	*Arthrobacter*
	Cha37	+	+	−	−	−	+	−	+	+	−	−	+	−	+	−	Rod and cocci	−	*Arthrobacter*
	Cha38	+	+	−	−	−	+	−	+	+	−	−	−	+	+	−	Rod and cocci	−	*Arthrobacter*
	Cha39	+	+	−	−	−	+	−	+	+	−	−	+	−	+	−	-	−	*Arthrobacter*
	Cha40	+	+	+	+	−	+	−	+	+	−	−	−	−	−	+	Minute cocci	−	*Micrococcus*
	Cha41	+	+	+	+	−	+	−	+	−	+	−	−	−	−	+	Minute cocci	−	*Micrococcus*
	Cha42	+	+	+	−	+	+	−	−	+	+	−	−	−	+	+	Medium rods	Central	*Bacillus*
	Cha43	+	+	+	−	+	+	−	−	+	+	−	−	+	+	+	Long rods	Central	*Bacillus*
	Cha44	−	+	+	+	+	−	+	+	+	−	+	+	+	+	−	Rod shape	−	*Pseudomonas*
	Cha45	+	+	−	−	−	+	−	+	+	−	−	−	+	+	−	Rod and cocci	−	*Arthrobacter*
	Cha46	+	+	−	−	−	+	−	+	+	−	−	+	−	+	−	-	−	*Arthrobacter*

In the current study, the strains were primarily screened for their ability to tolerate drought stress, while considering important physiological and biochemical traits relevant to plant growth promoters. In the absence of drought stress, phosphate-solubilizing activity corresponded with high amounts of dissolved phosphorus, which were caused by strains ZM4 (256 mg L^−1^), ZM18 (239 mg L^−1^), ZM44 (237 mg L^−1^), Cha13 (236 mg L^−1^) and Cha27 (235 mg L^−1^) (Table 3). Meanwhile, drought stress led to high amounts of dissolved phosphorous in strains ZM39 (248 mg L^−1^), ZM18 (254 mg L^−1^), Cha15 (241 mg L^−1^) and Cha43 (269 mg L^−1^). High amounts of siderophore production were recorded in strains Cha38 (24.5%), Haw25 (23.7%), ZM4 (22.7%) and Haw20 (21.4%) under normal conditions, whereas drought stress induced high levels of siderophore production by Cha43 (28.6%), ZM39 (28.4%), Haw25 (25.8%) and ZM4 (24.6%), respectively.

**Table 3:** Multiple plant growth promoting activities of the promising rhizobacterial strains, isolated from different rhizospheres of walnut trees, under control (0 MPa) and PEG_6000_-induced drought stress (− 1.5 MPa) treatment.

Rhizobacteria strains	Phosphate-solubilizing activity (mg L^−1^)		Siderophore production (% Siderophore unit)		IAA production (μg mL^−1^)		Gibberellic acid (GA_3_) production (μg mL^−1^)		HCN production	
	Control	− 1.5 MPa	Control	− 1.5 MPa	Control	− 1.5 MPa	Control	− 1.5 MPa	Control	− 1.5 MPa
ZM4	*256 ± 8.66	247 ± 6.11	22.71 ± 1.33	24.61 ± 1.77	21.33±1.55	22.52 ± 1.25	74.35 ± 6.44	79.45 ± 4.17	+++	++++
ZM7	213 ± 7.50	235 ± 10.42	19.53 ± 1.08	21.45 ± 2.08	22.70±1.04	24.61 ± 1.15	68.74 ± 7.11	75.62 ± 3.15	++	+++
ZM12	216 ± 5.43	248 ± 13.48	22.42 ± 1.80	22.70 ± 1.65	20.41±2.02	20.90 ± 2.09	64.22 ± 5.09	71.34 ± 5.21	+++	+++
ZM14	182 ± 8.00	196 ± 7.07	20.85 ± 2.01	21.90 ± 0.98	21.32±1.87	21.54 ± 1.01	69.25 ± 3.45	71.56 ± 4.89	++	+++
ZM18	239 ± 11.02	254 ± 8.51	19.31 ± 2.11	21.60 ± 0.54	20.80±0.09	21.62 ± 0.90	74.81 ± 7.00	82.54 ± 7.21	+++	+++
ZM26	207 ± 3.54	184 ± 4.00	21.31 ± 3.01	18.72 ± 1.54	19.80±0.90	20.18 ± 1.87	66.23 ± 4.50	74.90 ± 5.62	++	++++
ZM39	183 ± 6.62	248 ± 12.09	19.84 ± 1.84	28.43 ± 2.67	20.51±1.23	28.73 ± 2.93	69.74 ± 5.65	85.41 ± 6.43	++	+++
ZM44	237 ± 12.03	216 ± 11.17	17.22 ± 2.35	18.61 ± 2.01	21.38±2.32	22.61 ± 2.31	75.16 ± 4.45	77.80 ± 4.55	+++	+++
SS23	172 ± 8.34	163 ± 3.95	18.30 ± 1.02	19.24 ± 1.87	18.47±1.43	17.52 ± 1.95	52.35 ± 3.87	54.26 ± 3.54	+	+
SS212	192 ± 2.98	205 ± 8.61	16.45 ± 0.90	15.80 ± 1.03	17.66±1.09	16.35 ± 0.98	56.87 ± 2.54	53.67 ± 4.31	++	++
SS218	196 ± 5.27	185 ± 3.78	18.72 ± 1.21	17.90 ± 0.60	16.28±1.61	15.44 ± 1.31	59.39 ± 3.89	55.74 ± 4.00	+++	++
SS224	227 ± 9.56	213 ± 9.41	15.10 ± 2.01	14.70 ± 1.02	16.81±1.31	16.46 ± 1.23	69.73 ± 7.00	64.16 ± 3.43	++	++
TT24	162 ± 7.08	174 ± 7.34	16.81 ± 1.32	16.25 ± 0.89	15.72±0.90	15.90 ± 0.91	54.25 ± 3.71	52.72 ± 5.45	+++	++
TT211	186 ± 3.65	190 ± 12.03	18.73 ± 2.30	19.16 ± 1.32	18.33±2.01	18.18 ± 1.06	61.24 ± 4.57	58.90 ± 3.67	++	++
TT213	215 ± 6.76	206 ± 13.23	16.90 ± 0.89	15.43 ± 1.01	16.80±1.65	17.33 ± 0.08	57.36 ± 4.61	58.91 ± 3.76	+++	++
TT222	194 ± 4.87	187 ± 6.83	14.72 ± 0.08	13.51 ± 0.34	16.90±1.29	16.47 ± 1.01	61.80 ± 7.11	59.64 ± 2.98	+	+
Haw7	219 ± 10.16	236 ± 12.43	18.90 ± 1.01	21.70 ± 0.98	20.70±0.98	23.57 ± 2.21	74.25 ± 6.19	80.55 ± 6.98	+	+++
Haw14	201 ± 8.56	227 ± 14.01	17.54 ± 2.06	19.32 ± 1.05	21.72±2.04	25.90 ± 2.78	77.91 ± 4.76	82.42 ± 7.00	++	++++
Haw20	193 ± 4.76	204 ± 5.87	21.43 ± 1.90	23.90 ± 2.03	22.53±2.23	23.72 ± 2.04	80.92 ± 8.31	86.70 ± 6.39	++	++
Haw25	199 ± 6.68	202 ± 6.72	23.72 ± 2.31	25.80 ± 1.95	21.54±1.51	24.75 ± 1.65	76.26 ± 2.99	81.68 ± 4.57	+++	++
TT17	154 ± 5.38	135 ± 2.98	15.25 ± 0.06	14.13 ± 1.03	18.32±2.03	17.47 ± 2.00	54.34 ± 3.21	52.73 ± 2.93	++	++
TT110	142 ± 7.98	137 ± 5.61	18.20 ± 0.09	17.44 ± 2.01	20.52±1.94	19.15 ± 1.84	56.82 ± 4.23	58.47 ± 5.54	+	++
Cha15	218 ± 10.23	241 ± 12.32	20.51 ± 1.03	23.75 ± 2.21	22.71±2.98	24.66 ± 2.90	76.91 ± 5.32	82.74 ± 6.83	++	+++
Cha13	236 ± 13.04	242 ± 10.92	19.62 ± 2.03	21.32 ± 1.98	21.54±1.32	24.80 ± 2.43	69.46 ± 4.55	75.66 ± 6.00	+	+++
Cha21	202 ± 11.51	219 ± 3.98	18.60 ± 1.06	21.21 ± 2.00	24.13±2.04	26.90 ± 1.09	58.35 ± 2.99	65.24 ± 4.56	++	+++
Cha38	198 ± 6.80	207 ± 4.49	24.51 ± 2.40	25.70 ± 2.92	21.30±1.54	23.73 ± 1.02	68.44 ± 5.32	73.90 ± 6.43	++	++
Cha43	217 ± 5.62	269 ± 14.05	20.1 ± 1.21	28.60 ± 1.39	22.20±2.61	29.44 ± 2.13	69.71 ± 4.51	72.51 ± 4.43	+	++++
Cha41	183 ± 5.71	191 ± 10.11	19.35 ± 0.90	21.43 ± 0.09	24.72±2.47	25.46 ± 2.02	72.83 ± 5.68	94.32 ± 6.98	++	+++
Cha19	213 ± 9.81	227 ± 11.81	18.44 ± 2.03	20.16 ± 1.03	20.38±1.76	22.51 ± 1.99	65.15 ± 4.15	69.76 ± 7.21	+	++
Cha27	235 ± 4.87	248 ± 7.91	21.52 ± 1.98	23.71 ± 2.31	21.71±2.03	24.83 ± 2.01	60.73 ± 5.21	71.61 ± 5.29	++	++
Cha28	187 ± 8.31	201 ± 6.89	19.23 ± 0.39	20.62 ± 1.48	19.62±1.94	20.74 ± 0.98	78.91 ± 6.89	84.50 ± 5.81	++	+++
**LSD	25.83		5.65		4.94		17.83			

*Value represent mean ± SEM; ** Least Significant Difference (LSD; p < *0.001)* for mean comparison of strain-drought interaction effects.

The ability of microbes to release metabolites, such as organic acids, can be used as an index for determining phosphate-solubilizing activity^23^. The ability of bacteria to secrete organic acids is a function of mechanisms that are controlled by bacterial gene expression patterns which, in turn, can be influenced by environmental factors. Many phosphate-solubilizing bacteria (PSB) can forage Fe from the mineral complex into soluble Fe^3+^ which takes form through mechanisms of active transport carriers^24^. Siderophore production by PSB could improve the availability of P indirectly. Since siderophores are ligands that can extract Fe from ferric phosphate and ferric citrate^25^, PSBs tend to produce organic acid compounds that help to transform metal species into chelates, thereby reducing metal toxicity^26^.

The ability of plants to produce auxin is one of the main traits by which plant growth promoters are generally measured. Auxins are a group of hormones which act as molecular signals that regulate plant growth, prolong cell proliferation, cell division and differentiation. While coexisting with plants, different bacteria produce various amounts of auxin and release them into the rhizosphere. Auxins are reportedly produced by *Rhizobium*, *Bradyrhizobium* and *Nostoc* species, as well as by other species which occur in the rhizosphere^27^. Among the strains in the current research, the ability to produce auxins differed significantly among the bacterial species. *Bacillus* strains Cha41 (24.7 μg mL^−1^), Cha21 (24.1 μg mL^−1^) and ZM7 (22.7 μg mL^−1^) had the highest performance in producing auxin under normal conditions. Under drought stress, however, IAA formation increased in the *Bacillus* strains Cha43 (29.4 μg mL^−1^), ZM39 (28.7 μg mL^−1^) and Cha21 (26.9 μg mL^−1^) (Table 3). The current results are in agreement with those previously reported by Beneduzi et al.^28^ regarding the ability of *Bacillus* strains to produce auxin in Luria and Berthani Bruce media. Similarly, Lwin et al.^29^ and Kaur and Sharma^30^ showed that rhizobacteria produced auxin (53.1–71.1 μg mL^−1^) under optimal growth conditions, whereas Husen et al.^31^ reported lower amounts of bacterial auxin production (33.28 μg mL^−1^). Shobha and Kumudini^32^ reported a wide range of IAA (35–217 μg mL^−1^) produced by bacteria, since IAA production by PGPB strains can be affected by different factors, including the species of the microorganisms, the conditions in which plants and bacteria coexist, the specificity of each growth stage in plants, and the availability of suitable substrates^9,33^.

The current study showed significant differences in the amounts of GA_3_ which were produced by the different strains. The highest gibberellin production was observed in strain Haw20 (80.9), followed by Cha28 (78.9) and Haw14 (77.9) (μg mL^−1^). In contrast, drought-stressed strains produced significantly higher amounts of GA_3_, as observed in Cha41 (94.3), Haw20 (86.7) and ZM39 (85.4) (μg mL^−1^) (Table 3). Previous studies reported that the maximum amount of gibberellin (65.3 μg mL^−1^) was produced by *Pseudomonas* sp. when the bacteria were isolated from the wastes of processed olive fruits in a culture medium of NB^34^.

**Figure 1:**
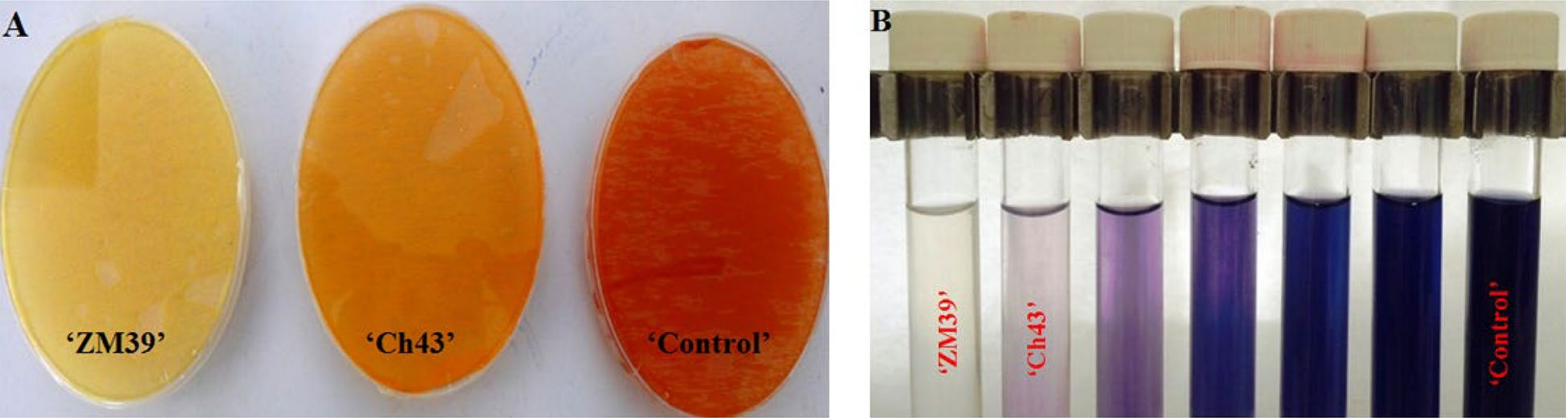
Color-based method for hydrogen cyanide (HCN) quantification in agar medium at the present of strains ‘Zm39’, ‘Ch43’, and no-strain (‘Control’) **(A)**, and starch hydrolysis method for assaying the amylase activity **(B)** under drought stress induced by PEG_6000_.

One of the secondary metabolites produced by the PGPR is hydrogen cyanide, which plays an important role in the biological control of pathogens. In this study, all of the evaluated strains produced HCN, although their levels of production were not similar. Strains ZM39 and Cha43 produced the highest amounts of HCN, so much that the color of filter papers changed from pale yellow to brown. Also, these two strains showed starch hydrolysis activity along with other 134 strains (Fig. 1). Earlier research in the available literature suggested that HCN production by PGPR can promote plant growth by inactivating pathogens, but recent findings have argued that HCN indirectly increases P availability by metal chelation^35^. Increasing of soluble sugar contents in the leaves of *Brachypodium distachyon* (switchgrass) treated by some *Bacillus* strains has been reported^39^.

Based on morphological, biochemical and biological assessments, while considering plant response to drought stress, two promising strains were identified, ZM39 and Cha43, which caused high levels of resistance to drought. Thus, both strains were selected for further genetic identification by molecular markers. Upon completing the amplification and sequencing of their 16S rDNA gene sequences, and after using the BLAST-N program (NCBI), a complete identification showed > 99% similarity of their partial sequence with the available sequences from the NCBI database. The obtained sequences were submitted to NCBI and, together with other relevant information, their accession numbers were provided (Table 4). According to molecular assessments, ZM39 and Cha43 strains were identified as members of *B. velezensis* and *B*. *amyloliquefaciens*, respectively (Table 4). The current research identified these two rhizobacterial species, through DNA molecular analysis, in walnut rhizospheres for the first time. Previous reports indicated the presence of *Azotobacter, Azospirillum, Bacillus, Pseudomonas, Aspergillus* and *Penicillium* genera in walnut rhizospheres^7^.

**Table 4:** Molecular characterization of two selected rhizobacteria isolates based on16s rDNA sequencing.

Rhizobacteria isolates	Accession number at NCBI	No. of amplified base pairs	Isolates identification	Forward and reverse primers
‘ZM139’	MK757975	530	*Bacillus velezensis*	F: 5′-AGAGTTTGATCTTGGCTCAG-3′ R:5′-AAGGAGGTGATCCAGCCGCA-3′
‘Cha43’	MK757976	534	*Bacillus amyloliquefaciens*	F: 5′-AGAGTTTGATCTTGGCTCAG-3′ R:5′-AAGGAGGTGATCCAGCCGCA-3′

Large number of strains of *Bacillus spp*. have been isolated from and characterized in relation to plants. Recent phylogenomic studies have demonstrated that *Bacillus methylotrophicus*, *Bacillus velezensis, Bacillus oryzicola* and *Bacillus amyloliquefaciens* subsp. *plantarum* can be considered as heterotypic synonyms^36^. Among the strains of the *B. velezensis* clade, secondary metabolites are diversely created by bacterial cells, which could exhibit antibacterial and anti-stress activities^37^. In the current study, with respect to the 16S rDNA gene sequence, phenotypic and molecular characteristics of *B. velezensis* were similar to those of *B. amyloliquefaciens*. In previous research, *gyrB* gene sequences confirmed that *B. velezensis* and *B. amyloliquefaciens* had similarities in heterotypic terms^38^.

Strains of the *Bacillus* genus have reportedly enhanced drought-tolerance in switchgrass through upregulation of drought-responsive genes and the modulation of the DNA methylation process^39^. Other strains can also make close associations with host plants and produce phytohormones, along with several well-characterized lipopeptide toxins^40^. These characteristics suggest that these strains have strong potential to act as bio-inoculants and can increase biomass in fruit trees, while assisting the defense system in plants against abiotic stress. PGPR can ultimately contribute to the production of plant growth regulators such as gibberellins^41^, auxins^42,43^, cytokinins and ABA^44^, thereby mitigating the adverse effects of abiotic stress on the physiological and biochemical processes of plants. Hormone levels in plant tissues could be modulated by microbial regulators via mechanisms that mimic the modes of exogenous phytohormone application^45,46^.

## Conclusion

A large outlook of research potential is envisaged to explore walnut trees in terms of microbial populations in their rhizospheres. In this work, a significant diversity of PGPRs was confirmed in walnut rhizospheres. In addition, drought stress induced the ability of the identified PGPRs to solubilize phosphates and produce siderophore, IAA, gibberellic acid and HCN. Thus, there is scope that these PGPRs can be used as bio-fertilizers for sustainable crop production. Such improvements in the capabilities of plants may protect them against various forms of biotic and abiotic stress. Two promising strains (ZM39 and Cha43) were identified based on the current morpho-biochemical assays upon drought stress treatment. These strains were molecularly identified as *B. velezensis* and *B*. *amyloliquefaciens*, respectively. An ongoing project involves specifying their roles in improving tolerance against drought stress in walnut seedlings.

## Supplementary Information

Supplementary Information.

## Data Availability

Sequencing-data were generated during the current study and are available at NCBI as follows: *Bacillus velezensis* strain ZM39 16S ribosomal RNA gene, partial sequence; GenBank No. MK757975.1 (https://www.ncbi.nlm.nih.gov/nuccore/MK757975.1/). *Bacillus amyloliquefaciens* strain Cha43 16S ribosomal RNA gene, partial sequence; GenBank No. MK757976.1 (https://www.ncbi.nlm.nih.gov/nuccore/MK757976.1/).

## References

[1] Hartman K, Tringe SG (2019). Interactions between plants and soil shaping the root microbiome under abiotic stress. Biochem. J..

[2] Liu FC (2014). Effects of inoculating plant growth-promoting rhizobacteria on the biological characteristics of walnut (*Juglans regia*) rhizosphere soil under drought condition. J. Appl. Ecol..

[3] Yu X, Liu X, Zhu TH, Liu GH, Mao C (2012). Co-inoculation with phosphate-solubilzing and nitrogen-fixing bacteria on solubilization of rock phosphate and their effect on growth promotion and nutrient uptake by walnut. Eur. J. Soil Biol..

[4] Ruiz-Lozano JM, Porcel R, Azcón C, Aroca R (2012). Regulation by arbuscular mycorrhizae of the integrated physiological response to salinity in plants: New challenges in physiological and molecular studies. J. Exp. Bot..

[5] Parisa M, Tozlu E, Kotan R, Kotan MŞ (2017). Potential of some bacteria for biological control of postharvest citrus green mould caused by *Penicillium digitatum*. Plant Protec. Sci..

[6] Gururani MA, Upadhyaya CP, Strasser RJ, Woong YJ, Park SW (2012). Physiological and biochemical responses of transgenic potato plants with altered expression of PSII manganese stabilizing protein. Plant Physiol. Biochem..

[7] Dar NA, Khan MA, Zargar MY (2009). Development of plant growth promoting rhizosphere microflora as inoculants for walnut (*Juglans regia *L.). Indian For..

[8] Shiwani GS, Sharma K, Walia A, Chauhan A, Shirkot CK (2014). Population and functional diversity of phosphate solubilizing bacteria from apricot (*Prunus armeniaca*) of mid and high regions of Himachal Pradesh. Bioscan.

[9] Hassan Dar GH, Sofi S, Padder SA, Kabli A (2018). Molecular characterization of rhizobacteria isolated from walnut (*Juglans regia*) rhizosphere in Western Himalayas and assessment of their plant growth promoting activities. Biodiversitas.

[10] Dipta B, Kirti S, Bhardwaj S, Gupta S, Kaushal R (2017). Phosphate solubilizing potential of *Bacillus pumilus* for the enhancement of Cauliflower (*Brassica oleracea* var. botrytis L.). Ecol. Environ. Cons..

[11] Yu X, Liu X, Zhu TH, Liu GH, Mao C (2011). Isolation and characterization of phosphate-solubilizing bacteria from walnut and their effect on growth and phosphorus mobilization. Biol. Fertil. Soils..

[12] Behrooz A (2019). Arbuscular mycorrhiza and plant growth-promoting bacteria alleviate drought stress in walnut. HortScience.

[13] Martínez-Viveros O, Jorquera MA, Crowley DE, Gajardo G, Mora ML (2010). Mechanisms and practical considerations involved in plant growth promotion by rhizobacteria. J. Soil Sci. Plant Nutr..

[14] Bergey DH, Holt JG (2000). Bergey's Manual of Determinative Bacterology.

[15] Sperber JI (1958). Solution of apatite by soil microorganisms producing organic acids. Aust. J. Agric. Res..

[16] Nack-Moon S, Kim K, Choi SH (1993). Isolation of soil bacteria secreting raw-starch-digesting enzyme and the enzyme production. J. Micro Biotech..

[17] Schwyn B, Neilands JB (1987). Universal chemical assay for the detection and determination of siderophores. Anal. Biochem..

[18] Lukkani NJ, Reddy ECS (2014). Evaluation of plant growth promoting attributes and biocontrol potential of native fluorescent *Pseudomonas* spp. against Aspergillus niger causing collar rot of ground nut. Int. J. Plant Anim. Environ. Sci..

[19] Żur I (2015). Hormonal requirements for effective induction of microspore embryogenesis in triticale (×*Triticosecale Wittm*.) anther cultures. Plant Cell Rep..

[20] Ubaidillah M, Safitri FA, Lee S, Park GH, Kim KM (2015). Alteration of plant hormones in transgenic rice (*Oryza sativa* L.) by overexpression of anti-apoptosis genes during salinity stress. J. Plant Biotech..

[21] Ruzzi M, Aroca R (2015). Plant growth-promoting rhizobacteria act as biostimulants in horticulture. Sci. Hortic..

[22] Vega FE, Pava-Ripoll M, Posada F, Buyer JS (2005). Endophytic bacteria in *Coffea arabica* L. J. Basic Microbiol..

[23] Khan MS, Ahmad E, Zaidi A, Oves M, Maheshwari DK, Saraf M, Aeron A (2013). Functional aspect of phosphate-solubilizing bacteria: Importance in crop production. Bacteria in Agrobiology: Crop Productivity.

[24] Collavino MM, Sansberro PA, Mroginski LA, Aguilar OM (2012). Comparison of in vitro solubilization activity of diverse phosphate-solubilizing bacteria native to acid soil and their ability to promote *Phaseolus vulgaris* growth. Biol. Fertil. Soils.

[25] Zaidi A, Khan MS, Ahemad M, Oves M (2009). Plant growth promotion by phosphate solubilizing bacteria. Acta Microbiol. Immunol. Hung..

[26] Ahemad M (2015). Phosphate-solubilizing bacteria-assisted phytoremediation of metalliferous soils: A review. 3 Biotech..

[27] Noor Ai’shah O (2013). Influence of indole-3-acetic acid (IAA) produced by diazotrophic bacteria on root development and growth of in vitro oil palm shoots (*Elaeis guineensis* Jacq.). J. Oil Palm Res..

[28] Beneduzi A, Peres D, Vargas LK, Bodanese-Zanettini MH, Passaglia LMP (2008). Evaluation of genetic diversity and plant growth promoting activities of nitrogen-fixing bacilli isolated from rice fields in South Brazil. Appl. Soil Ecol..

[29] Lwin KM, Myint MM, Tar T, Aung WZM (2012). Isolation of plant hormone (indole-3-acetic acid-IAA) producing rhizobacteria and study on their effects on maize seedling. Eng. J..

[30] Kaur N, Sharma P (2013). Screening and characterization of native Pseudomonas sp. as plant growth promoting rhizobacteria in chickpea (*Cicer arietinum* L.) rhizosphere. Afr. J. Microbiol. Res..

[31] Husen E (2003). Screening of soil bacteria for plant growth promotion activities in vitro. Indones. J. Agric. Sci..

[32] Shobha G, Kumudini B (2012). Antagonistic effect of the newly isolated PGPR *Bacillus* spp. on *Fusarium oxysporum*. Int. J. Appl. Sci. Eng..

[33] Kannahi M, Kowsalya M (2013). Efficiency of plant growth promoting rhizobacteria for the enhancement of *Vigna mungo* growth. J. Chem. Pharm. Res..

[34] Karakoç Ş, Aksöz N (2006). Some optimal cultural parameters for gibberellic acid biosynthesis by *Pseudomonas *sp. Turk. J. Biol..

[35] Rijavec T, Lapanje A (2016). Hydrogen cyanide in the rhizosphere: Not suppressing plant pathogens, but rather regulating availability of phosphate. Front. Microbiol..

[36] Dunlap CA, Kim SJ, Kwon SW, Rooney AP (2016). *Bacillus velezensis* is not a later heterotypic synonym of *Bacillus amyloliquefaciens*; *Bacillus methylotrophicus*, *Bacillus amyloliquefaciens* subsp. plantarum and *Bacillus oryzicola* are later heterotypic synonyms of *Bacillus velezensis* based on phylogenomics. Int. J. Syst. Evol..

[37] Palazzini JM (2016). Biological control of *Fusarium graminearum sensu stricto*, causal agent of Fusarium head blight of wheat, using formulated antagonists under field conditions in Argentina. Biol. Control.

[38] Liu H, Wu XQ, Ren JH, Ye JR (2011). Isolation and identification of phosphobacteria in poplar rhizosphere from different regions of China. Pedosphere.

[39] Gagne-Bourque F (2015). Accelerated growth rate and increased drought stress resilience of the model grass *Brachypodium distachyon* colonized by *Bacillus subtilis* B26. PLoS ONE.

[40] Gagne-Bourgue F (2013). Isolation and characterization of indigenous endophytic bacteria associated with leaves of switchgrass (*Panicum virgatum* L.) cultivars. J. Appl. Microbiol..

[41] Khan MIR, Asgher M, Khan NA (2014). Alleviation of salt-induced photosynthesis and growth inhibition by salicylic acid involves glycinebetaine and ethylene in mungbean (*Vigna radiata* L.). Plant Physiol. Biochem..

[42] Etesami H, Alikhani HA, Hosseini HM (2015). Indole-3-acetic acid (IAA) production trait, a useful screening to select endophytic and rhizosphere competent bacteria for rice growth promoting agents. MethodsX.

[43] Pereira SIA, Monteiro C, Vega AL, Castro PML (2016). Endophytic culturable bacteria colonizing *Lavandula dentata* L. plants: Isolation, characterization and evaluation of their plant growth-promoting activities. Ecol. Eng..

[44] Kudoyarova GR (2014). Cytokinin producing bacteria stimulate amino acid deposition by wheat roots. Plant Physiol. Biochem..

[45] Shahzad R (2016). Seed-borne endophytic *Bacillus amyloliquefaciens* RWL-1 produces gibberellins and regulates endogenous phytohormones of *Oryza sativa*. Plant Physiol. Biochem..

[46] Turan M (2014). Plant growth-promoting rhizobacteria improved growth, nutrient, and hormone content of cabbage (*Brassica oleracea*) seedlings. Turk. J. Agric. For..

