# Supplementary material for: Characterization of plant growth-promoting rhizobacteria (PGPR) in Persian walnut associated with drought stress tolerance
Source: Sci Rep. 2022 Jul 26;12:12725. doi: 10.1038/s41598-022-16852-6 (PMC9325983; doi:10.1038/s41598-022-16852-6)
Supplement: Supplementary file 1 — Supplementary Information. [file 41598_2022_16852_MOESM1_ESM.docx]

| **Supplementary table 1.** Morphological and phenological traits of the studied genotypes (adopted from lotfi et al., 2019). | | | | | | |
| --- | --- | --- | --- | --- | --- | --- |
| **Walnut genotypes** | **Leafing date** | **Tree Vigor** | **Dichogamy** | **Seed size (g)** | **Kernel percent** | **Yield** |
| **‘ZM1’** | Early | High | Protandrous | Medium (10-12) | >55 | Medium |
| **SS2’** | Early | Medium-low | Protandrous | Medium (10-12) | 45-50 | Medium-Low |
| **‘TT1’** | Early | Very High | Protandrous | Very big (>16) | 50-55 | Medium-Low |
| **‘TT2’** | Early | Medium | Protandrous | Big (12-14) | 45-50 | High |
| **‘Chandler’** | Late | Low | Protandrous | Medium (10-12) | 40-45 | Very high |
| **‘Haward’** | Late | High-medium | Protandrous | Medium (10-12) | 40-45 | Very high |
